# Supplementary material for: Landmark-based morphometrics with fewer landmarks: Some examples for medical entomology
Source: PLoS Negl Trop Dis. 2026 Jun 2;20(6):e0014386. doi: 10.1371/journal.pntd.0014386 (PMC13249138; doi:10.1371/journal.pntd.0014386)
Supplement: Attachment — Submitted filename: Responses to reviewers.pdf [file pntd.0014386.s003.pdf]

Additional Editor Comments:

Please strengthen the methodological clarity by specifying data sources, …

The data used were the same as those leading to the various publications listed in Table 1. As we were co-authors of these publications, the raw data are in our possession.

… software workflows,

All calculations were performed using the XYOM software except the resistant fit method (RF) of the Procrustes superposition, which used the R software.

… **and adding statistical significance tests for your key comparisons.**

The objective of the study was to show that similar or higher classification scores were possible with less landmarks, and when possible, it was not to show if any possible improvement was statistically significant.
Thus, instead of presenting possible statistics comparing two reclassification scores which could be themselves not significant, we used validated scores (Klecka, 1980). For instance, when reclassifying individuals between two groups having the same number of individuals, each reclassified individual has 50% of being correctly assigned, just by chance. The removal of this chance is possible, as suggested by Klecka (1980). The XYOM/miscellaneous script uses these "adjusted scores", which can be seen as scores answering the question: "how far does the observed score improve the one expected by chance only?"
Note that the publications from which the raw data were used did not apply any such correction or adjustment for chance. Our XYOM script performing the "random search" computes both the scores and the adjusted ones.
Using both kinds of score estimations, our study on influential landmarks produced the same general result: for some configurations, less landmarks were able to produce equal or higher (adjusted) scores.

Additionally, revise the manuscript to better contextualise the public health relevance of your findings and explicitly discuss the study's limitations.

The public health relevance of the finding is not different from the one generally admitted for the morphometric approach: a powerful complement to morphological identification, especially for distinguishing cryptic species. But the method is frequently considered as a laborious one because there is generally no automated tool allowing a reliable landmark digitization.
We added a paragraph dedicated to the public health relevance of the study, entitled "Epidemiological importance", also we added a paragraph about the "Limitations of the study".

Responses to reviewers

First of all, we would like to sincerely thank both reviewers for their patient work and helpful comments or questions.

#1

Title
The current title, “Landmark-based geometric morphometrics: Influential landmarks,” effectively reflects the methodological focus of the study. However, could the authors consider revising or expanding the title to highlight its potential contribution to species discrimination in medically important or vector insects, to better align with the journal’s applied scope?

We suggest the following:
Landmark-based morphometrics with fewer landmarks: some examples for medical entomology.

Abstract
The abstract effectively summarizes the overall scope of the study. Nevertheless, it could be strengthened by adding essential statistical results or numerical findings that support the main conclusions, ensuring the abstract fully reflects the study’s analytical depth.

The abstract was modified (see file “Revised manuscript with track changes”) with the hope to be better than the first one.
The objective was to determine whether it was possible to equal, or even surpass, the classification scores obtained with a reduced number of anatomical landmarks. Instead of presenting possible statistics comparing two reclassification scores which could be themselves not significant, we propose to recompute all the examples using scores as adjusted for chance (Klecka, 1980)

Introduction
1. In lines 2–3, the introduction briefly mentions that species identification can be difficult for morphologically similar or cryptic taxa. However, it does not sufficiently address why successful discrimination of these species is biologically or epidemiologically important, particularly in the context of vector-borne disease transmission. To better align with the scope of PLOS Neglected Tropical Diseases, the authors should elaborate on the biological relevance and public health implications of accurately identifying cryptic or closely related vector species. For instance, how would improved morphological discrimination contribute to vector surveillance, disease risk mapping, or control strategies?

We added a brief section about the epidemiological relevance and public health implications of our findings. The revised text explains how identifying reduced but highly informative landmark subsets may improve the speed, reproducibility, and operational feasibility of morphometric identification for medically important vectors (see file “Revised manuscript with track changes”).

2. In lines 18–25, the authors attempt to describe the research gap related to the dimensionality of landmark-based analyses and refer to concepts such as first PCs, multidimensionality problems, and eigenvalues of the correlation matrix. However, these technical terms are introduced without sufficient explanation or context. Could the authors provide brief clarifications or background descriptions for these terms? For example, explain where the first principal components (PCs) come from, what the multidimensionality problem represents in geometric morphometrics, final shape variables, …

We modified the text as follows (see file "Revised manuscript with track changes"):

A larger number of landmarks**, each one represented by two variables (the x,y coordinates)** may result in too many shape variables to describe few individuals. As an acceptable solution to possible multidimensionality problems, **(as for instance an overfitting effect),** a subset of few first **principal components (**PCs**)** of the shape variables have commonly been used. **The principal component analysis (PCA) based on the covariance matrix is the basic multivariate analysis applied in geometric morphometrics. It generates new variables (the principal components, called here "final shape variables") ordered according to their respective contribution (their "eigenvalue") to the total variation. It allows for an important reduction of dimensionality, with often the two or three first PCs representing the main trends of differentiatio**n.

… and how eigenvalues in the correlation matrix are used to assess redundancy among landmarks.

This statistical approach for reduction of dimensionality, using the eigenvalues of a PCA based on a correlation matrix, has been used once in the past. We did not use it in our study but we mention it because as far as we know it was perhaps the very first study to try giving different values to different landmarks.

3. In the final sentence, lines 29–32, of the Introduction, the authors summarize their findings by stating that smaller subsets of carefully chosen landmarks can provide better taxonomic discrimination than full landmark sets. However, such a statement presents results or conclusions that belong more appropriately in the Results or Discussion section rather than in the Introduction. It would be more suitable here to focus on the potential benefits or significance of the study instead.

We removed this statement and replaced it with the following sentence:
" **Accurate species identification is essential during the monitoring phase of vector control programs. In addition to the requirement for accuracy, the identification method must be inexpensive and readily available. The morphometric approach offers these advantages without requiring extensive entomological expertise. However, its use as a routine technique could be hampered by the difficulty of data collection. Digitizing many landmarks can be a lengthy and tedious process, deterring many entomologists working with large samples. This drawback has likely hindered the adoption of**

geometric morphometry in many laboratories. Therefore, any idea that reduces the workload without compromising discriminatory power is welcome.“

Materials and Methods

1. In lines 35–92, the manuscript does not clearly specify the sources of the morphometric data used in each analysis. It is unclear whether the landmark coordinates were newly digitized from specimens or re-used from previously published datasets or online repositories.

We added the following to the “**Qualifying landmarks**” section (see file “Revised manuscript with track changes”)
“The data used on our material were the raw coordinates obtained by the authors of the publications listed in Table 1. Our reclassification of the species, however, used a different algorithm than the linear discriminant analysis (LDA), i.e., the K-means algorithm. We validated our reclassification scores by adjusting for any correct assignment that would be obtained by chance (see ``Classification method" in this section, as well as ``Classification tool" in the ``Discussion" section).”

However, i note that *Table 1 (“Materials. Genera and Species. , not mentioned; op, operator; F, females; M, males; peudow., pseudowillmori; sawad., sawadwongporni”) appears in the Results section. Given that the study analyzes images/data derived from previously published sources (i.e., secondary material), this table belongs more appropriately in the Materials and Methods section.

The Table appears in the Result section because I could not control its spatial position in the final pagination (automatically assigned by the latex software). But its legend starts with the word “Materials”, suggesting that it is indeed related to the “Materials and Methods” section.

2. In lines 93–106, Several technical terms used in the Materials and Methods section, such as

…Procrustes superposition, resistant-fit (RF), …
See changes at the “Qualifying landmarks” section of the “Revised manuscript with track changes” file)
“Using two species from each family, we compared the reclassification accuracy obtained with the total number of landmarks and the ones obtained with successively lower numbers of landmarks. Thus, we built different subsets of landmarks, decreasing from the total set of landmarks up to a minimum subset of 3 landmarks.
For each subset of landmarks, we performed the Generalized Procrustes Analysis (GPA): **it refers to the superposition of at least two configurations of homologous coordinates, thus corresponding to at least two different individuals. This is done by optimally aligning their respective coordinate systems, producing “aligned” configurations. Optimality can be achieved parametrically, using the least squares (LS) method, or nonparametrically, using the resistant fit (R) method. In this process, each**

configuration is assigned the same unit of size by dividing its x,y coordinates by the centroid size. Detailed explanation is available in …"

… allometric residue, …
see the "Revised manuscript with track changes" file), added text is:
"Since the shape variables are unlikely to completely remove the possible influence of size variation (allometry), for each pairwise comparison we computed an estimate of this possible effect as the coefficient of determination of size variation on shape variation (the residual allometry)}. We then could compute the correlation between the allometric residue of shape for each Ra subset and the accuracy score it allowed. The idea was to detect a possible interference of size on the level of reclassification accuracy, trying to answer the question: ``does the level of residual allometry determine the level of accuracy?''}. We also computed the correlation between the accuracy scores of the subsets and the corresponding Procrustes distances, expecting a likely high positive correlation.

… random search method, and hierarchical method, are introduced without sufficient explanation or definition.
These methods had each one a separate sub-section for detailed description in the MATERIALS AND METHODS section, as well as two separate sub-sections in the DISCUSSION section.
Since the referee does not indicate where there is a special need for complementary explanations, I do not know how to modify the first draft.

For clarity and accessibility, it is recommended that the authors provide brief descriptions or conceptual explanations of these terms when they first appear. This will help readers—especially those less familiar with geometric morphometric techniques—understand the analytical framework and rationale behind each procedure more clearly.

Suggested changes

Qualifying landmarks l. 93
Using two species from each family, we compared the reclassification accuracy obtained with the total number of landmarks and the ones obtained with successively lower numbers of landmarks. Thus, we built different subsets of landmarks, decreasing from the total set of landmarks up to a minimum subset of 3 landmarks.
For each subset of landmarks, we performed the Generalized Procrustes Analysis (GPA)**: it refers to the superposition of at least two configurations of homologous coordinates, thus corresponding to at least two different individuals. This is done by optimally aligning their respective coordinate systems, producing "aligned" configurations. Optimality can be achieved parametrically, using the least squares (LS) method, or nonparametrically, using the resistant fit (R) method. In this process, each configuration is assigned the same unit of size by dividing its x,y coordinates by the centroid size. Detailed explanation is available in** …

We used an estimate of the Procrustes distance, i.e., the distance **separating aligned configurations** to re-classify the compared groups.

To give each **configuration** of landmarks an estimation of its
contribution to the discrimination of two groups, two main methods were applied.

Classification tools l.132
The reclassification of the samples was based on shape variables, not on size. Shape variables were computed according to the generalized Procrustes analysis (GPA) [18]. **Shape differences are supposed to exclude size differences, but such exclusion is generally not complete. There is a more or less important residual influence of size of shape, that we call the "allometric residue": it was computed as the coefficient of determination of the regression of shape on size… and used for** subsequent correlation analyses.

3. The manuscript applies K-means clustering for group reclassification, rather than the more commonly used supervised methods such as Linear Discriminant Analysis (LDA) or Canonical Variate Analysis (CVA), which are standard in geometric morphometric studies. Could the authors clarify why an unsupervised classification approach (K-means) was chosen instead of a supervised one?

This choice was explained in the section "**Classification method**" l.268 Please refer to the lines 268 to 288 which were already dedicated to this question.

4. The manuscript indicates that all computations were performed using the XYOM software. However, it remains unclear whether XYOM can directly calculate residual allometry for subsequent correlation analyses, or if this step required additional processing using other software (e.g., R). Could the authors clarify whether XYOM itself was used to compute the allometric residuals, or if a separate platform or custom script was applied for this purpose? In addition, since the manuscript mentions that the resistant-fit alignment was performed using the R "opSup" function [21], please specify whether R was also used for other parts of the analysis (such as allometry correction or statistical correlation). Providing these details would enhance methodological transparency and reproducibility.

As stated in the abstract and in the lines 144 to 148 about the software used, all the statistical and geometric computations were performed using the XYOM software, except the RF method of alignment, which was indeed performed using the opSup R function. We added the following words in the "Software section":
"**..., including correlation, allometry, and distance tests after Procrustes superposition, as well as reclassifications based on the k-means algorithm**…"

Ideally, the response to these comments could also detail the XYOM scripts (inputs, outputs) used to develop the methods for identifying influential landmarks. However, this would make the article too cumbersome.

Reviewer #2: The objectives of the study are clearly articulated, the study design is appropriate, the population and sample size are suitable, and the statistical analyses are correctly applied to support the conclusions.

However, it would be helpful if the Materials and Methods section clarified the relationship between the “random search method” and the “hierarchical method”—specifically, whether it is necessary to perform one method before evaluating the other, or how exactly they are related in the workflow.

We added the following section to end the description these two methods (see the “revised manuscript with track changes” file)

“**Relationship between the “random search method” and the “hierarchical method**”

The two approaches use entirely different methods to select influential landmarks. They are not presented here as a two-step strategy for identifying relevant subsets, but rather to illustrate the unexpected limitations of an intuitive approach such as the "hierarchical method." The latter provides a single output: a list of landmarks sorted according to their contribution to the overall distance between shapes. From this list, the user then selects a subset and excludes the less relevant landmarks. However, selecting these relevant landmarks does not offer the highest level of discrimination, as demonstrated by the "random search" method.
The "random search" method does indeed find more propositions belonging to influential subsets and frequently provides higher-performing subsets. It generally includes some (or even all) of the influential LMs suggested by the hierarchical method.

Results

Reviewer #1:

1. The results section mainly presents percentages and correlation coefficients; however, there is no indication of statistical significance testing (e.g., p-values).

Fixed

Could the authors include appropriate significance tests to confirm whether the subsets of landmarks truly outperform the full landmark sets in a statistically significant way?

The objective of the study was to check whether a smaller number of landmarks could produce classifications scores equivalent or higher than with the total number of landmarks. Thus, instead of presenting possible statistics comparing two reclassification scores (themselves perhaps not “significant”), the score reclassifications themselves were statistically tested. For instance, when reclassifying individuals between two groups having

the same number of individuals, each reclassified individual had 50% of being correctly assigned. Just by chance. The XYOM/miscellaneous script uses “adjusted scores” (Klecka, 1980), i.e., a score answering the question: “what is the proportion of correct assignments in addition to the ones that would be due to chance alone”.

2. All tables could be improved for better readability, as they currently contain a large number of abbreviations that may make interpretation difficult. I recommend revising the tables—especially the column headers—by avoiding abbreviations and using full terms instead. This would make the presentation clearer and more reader-friendly across all tables.

I apologize for the sometimes awkward presentation of some tables. They were difficult to read because the legend was incomplete and did not specify all the abbreviations (column headings). The legends are now complete.
I kept the abbreviations because I preferred that the table not exceed the width of the page.

Reviewer #2: z

The results are clearly and completely presented, and the figures, tables, and images are of sufficient quality and clarity. The analysis matches the stated analysis plan.

Conclusions

Reviewer #1: The Discussion and conclusion sections are excellently written — it explains the results clearly and effectively connects them to potential applications. However, I suggest adding a brief paragraph that highlights how these findings could be applied in vector surveillance and control programs, to better align the discussion with the journal's scope and readership.

We added the following paragraph: "Epidemiological importance" (please see the "revised manuscript with track changes" file)

"Accurate identification of insect vectors allows for mapping their geographic distribution and, above all, evaluating the effectiveness of control measures on target species. However, in vector control, precise species identification is not the only criterion to consider. Proof of this lies in the fact that the high precision of genetic identification techniques has not led to the abandonment of the classic morphological approach, based on dichotomous keys. The relevance of the traditional morphological approach remains due not only to its precision, but also to its low cost and immediate accessibility. These are valuable assets, particularly for entomological monitoring after a treatment campaign. Indeed, reinfestations by the target vector occur unpredictably, both in time and space; a reliable, rapid, and inexpensive identification technique is therefore essential. The limitations of the dichotomous key approach can arise with cryptic species or when the specimens examined are damaged. In these situations, geometric morphometry has proven to be a very useful complement. The geometric approach is also inexpensive, readily available, and, moreover, does not require advanced entomological expertise. Despite this, geometric morphometry remains underutilized in entomological laboratories. Its complexity may explain this. Manually digitizing numerous landmarks for each individual is tedious, and no reliable automated digitization technique has generated sufficient interest for widespread adoption. Any method that reduces the workload is welcome, and our study explored the possibility of achieving this by decreasing the number of landmarks without compromising discrimination capabilities. The combined use of dichotomous keys and a less cumbersome approach to geometric morphometry could thus significantly improve entomological monitoring programs."

Reviewer #2: The conclusions of the study are well supported by the data presented in the manuscript. However, the limitations of the analysis are not clearly described in a dedicated section or paragraph. Including a concise summary of the main limitations would provide important context and enhance the transparency and interpretability of the study's findings.

We redacted a new paragraph of the limitations of our study. Please see the "revised manuscript with track changes" file.

Limitations of our study

Our work is limited to the use of landmarks and does not concern semilandmarks (more frequent in three-dimensional morphometry), nor of course pseudolandmarks (the approach based on contours).
It empirically demonstrates the potential usefulness of more restricted landmark configurations for distinguishing related species. The results are based on the taxa, structures, and image dataset analyzed here, and the most informative landmark subsets may differ in other biological systems or applications. Indeed, our observation does not constitute mathematical proof, and we therefore cannot claim that it is valid for all other organisms. The benefit of a reduced number of landmarks should be validated in more detail on independent datasets and larger taxonomic groups before any large-scale operational implementation.
Although reducing the number of landmarks may improve efficiency, the quality of the digitization can still be influenced by specimen preservation, image resolution, and operator-related placement errors. Furthermore, reducing the number of reference points does not, in itself, solve the problem of collaboration between different operators, therefore it does not eliminate the need to work with data from a single human operator.
Finally, our proposal has not been field-tested to account for potential seasonal or geographic variations. Consequently, our results should be interpreted as demonstrating the potential of this optimization approach rather than defining a universally applicable strategy for reducing reference points.

Regarding the broader impact, the manuscript discusses how these data contribute to advancing our understanding of the studied technique, particularly in identifying efficient subsets of landmarks for morphometric analysis. However, the public health relevance of this work is not clearly detailed. While the introduction briefly mentions potential relevance, no further discussion is provided on this aspect in the results or discussion sections.

We added the following paragraph: “Epidemiological importance” (please see previous page or the “revised manuscript with track changes” file)

Summary and General Comments

Reviewer #1: -

Reviewer #2: This manuscript presents a well-structured study on the efficiency of landmark subsets for morphometric discrimination among closely related insect taxa. The objectives are clearly stated, the study design and statistical procedures are well detailed, the sample sizes are sufficient, and the statistical analyses are correctly applied. The results are clearly presented, and the figures and tables are of good quality. The conclusions are well supported by the data.

Among the strengths of the study are the systematic comparison of the random search and hierarchical methods, and the demonstration that smaller, carefully selected landmark subsets can, in some cases, outperform the full set. However, the relationship between the two methods is not fully explained, and the limitations of the

study are not explicitly presented. Additionally, while the technical utility of the findings is discussed, the public health relevance is only briefly mentioned and not explored in depth.
Overall, this is a novel and carefully executed study that advances our understanding of landmark selection in geometric morphometrics. Clarifying the relationship between the methods, explicitly stating the limitations, and contextualizing the relevance would further strengthen the manuscript.

We added the following paragraph: **“Relationship between the methods”** (please see the “revised manuscript with track changes” file)
The two approaches used entirely different methods to select influential landmarks. They were not presented here as a two-step strategy for identifying relevant subsets, but rather to illustrate the unexpected limitations of an intuitive approach such as the “hierarchical” method. The latter provides a single output: a list of landmarks sorted according to their contribution to the overall distance between shapes. From this list, the user then selects a subset and excludes the less relevant landmarks. However, selecting these relevant landmarks does not offer the highest level of discrimination, as demonstrated by the ”random search” method. The “random search” method does indeed find more propositions belonging to influential subsets and frequently provides higher-performing subsets. It may or not include some (or even all) of the influential landmarks suggested by the hierarchical method
