# Supplementary material for: Landmark-based morphometrics with fewer landmarks: Some examples for medical entomology
Source: PLoS Negl Trop Dis. 2026 Jun 2;20(6):e0014386. doi: 10.1371/journal.pntd.0014386 (PMC13249138; doi:10.1371/journal.pntd.0014386)
Supplement: Attachment — Submitted filename: renamed_488df.docx [file pntd.0014386.s001.docx]

**Reviewer’s comments**

This manuscript presents an analysis of landmark-based geometric morphometrics and challenges the classical assumption that increasing the number of landmarks improves shape discrimination. The concept of “influential landmarks” is highly relevant to morphometric research, particularly in entomological taxonomy and vector surveillance. Although the manuscript offers a novel and valuable contribution that deserves publication, certain methodological details require further clarification and elaboration to strengthen the overall study.

**Title**

The current title, “Landmark-based geometric morphometrics: Influential landmarks,” effectively reflects the methodological focus of the study. However, could the authors consider revising or expanding the title to highlight its potential contribution to species discrimination in medically important or vector insects, to better align with the journal’s applied scope?

**Abstract**

The abstract effectively summarizes the overall scope of the study. Nevertheless, it could be strengthened by adding essential statistical results or numerical findings that support the main conclusions, ensuring the abstract fully reflects the study’s analytical depth.

**Introduction**

1. In lines 2–3, the introduction briefly mentions that species identification can be difficult for morphologically similar or cryptic taxa. However, it does not sufficiently address why successful discrimination of these species is biologically or epidemiologically important, particularly in the context of vector-borne disease transmission. To better align with the scope of PLOS Neglected Tropical Diseases, the authors should elaborate on the biological relevance and public health implications of accurately identifying cryptic or closely related vector species. For instance, how would improved morphological discrimination contribute to vector surveillance, disease risk mapping, or control strategies?

2. In lines 18–25, the authors attempt to describe the research gap related to the dimensionality of landmark-based analyses and refer to concepts such as first PCs, multidimensionality problems, and eigenvalues of the correlation matrix. However, these technical terms are introduced without sufficient explanation or context. Could the authors provide brief clarifications or background descriptions for these terms? For example, explain where the first principal components (PCs) come from, what the multidimensionality problem represents in geometric morphometrics, final shape variables, and how eigenvalues in the correlation matrix are used to assess redundancy among landmarks.

3. In the final sentence, lines 29–32, of the Introduction, the authors summarize their findings by stating that smaller subsets of carefully chosen landmarks can provide better taxonomic discrimination than full landmark sets. However, such a statement presents results or conclusions that belong more appropriately in the Results or Discussion section rather than in the Introduction. It would be more suitable here to focus on the potential benefits or significance of the study instead.

**Materials and Methods**

1. In lines 35–92, the manuscript does not clearly specify the sources of the morphometric data used in each analysis. It is unclear whether the landmark coordinates were newly digitized from specimens or re-used from previously published datasets or online repositories. However, i note that *Table 1 (“Materials. Genera and Species. , not mentioned; op, operator; F, females; M, males; peudow., pseudowillmori; sawad., sawadwongporni”) appears in the Results section. Given that the study analyzes images/data derived from previously published sources (i.e., secondary material), this table belongs more appropriately in the Materials and Methods section.

2. In lines 93–106, Several technical terms used in the Materials and Methods section, such as Procrustes superposition, resistant-fit (RF), allometric residue, random search method, and hierarchical method, are introduced without sufficient explanation or definition. For clarity and accessibility, it is recommended that the authors provide brief descriptions or conceptual explanations of these terms when they first appear. This will help readers—especially those less familiar with geometric morphometric techniques—understand the analytical framework and rationale behind each procedure more clearly.

3. The manuscript applies K-means clustering for group reclassification, rather than the more commonly used supervised methods such as Linear Discriminant Analysis (LDA) or Canonical Variate Analysis (CVA), which are standard in geometric morphometric studies. Could the authors clarify why an unsupervised classification approach (K-means) was chosen instead of a supervised one?

4. The manuscript indicates that all computations were performed using the XYOM software. However, it remains unclear whether XYOM can directly calculate residual allometry for subsequent correlation analyses, or if this step required additional processing using other software (e.g., R). Could the authors clarify whether XYOM itself was used to compute the allometric residuals, or if a separate platform or custom script was applied for this purpose? In addition, since the manuscript mentions that the resistant-fit alignment was performed using the R “opSup” function [21], please specify whether R was also used for other parts of the analysis (such as allometry correction or statistical correlation). Providing these details would enhance methodological transparency and reproducibility.

**Results**

1. The results section mainly presents percentages and correlation coefficients; however, there is no indication of statistical significance testing (e.g., p-values). Could the authors include appropriate significance tests to confirm whether the subsets of landmarks truly outperform the full landmark sets in a statistically significant way?

2. All tables could be improved for better readability, as they currently contain a large number of abbreviations that may make interpretation difficult. I recommend revising the tables—especially the column headers—by avoiding abbreviations and using full terms instead. This would make the presentation clearer and more reader-friendly across all tables.

**Discussion**

The Discussion section is excellently written — it explains the results clearly and effectively connects them to potential applications. However, I suggest adding a brief paragraph that highlights how these findings could be applied in vector surveillance and control programs, to better align the discussion with the journal’s scope and readership.
