# Supplementary material for: Landmark-based morphometrics with fewer landmarks: Some examples for medical entomology
Source: PLoS Negl Trop Dis. 2026 Jun 2;20(6):e0014386. doi: 10.1371/journal.pntd.0014386 (PMC13249138; doi:10.1371/journal.pntd.0014386)
Supplement: Peer review history

# Peer review: Decision Letter 0

**DOI:** 10.1371/journal.pntd.0014386.r001

**Authors:** Clarence Mang'era

11 Feb 2026

PNTD-D-25-01192

Landmark-based geometric morphometrics: Influential landmarks

PLOS Neglected Tropical Diseases

Dear Dr. dujardin,

Thank you for submitting your manuscript to PLOS Neglected Tropical Diseases. After careful consideration, we feel that it has merit but does not fully meet PLOS Neglected Tropical Diseases's publication criteria as it currently stands. Therefore, we invite you to submit a revised version of the manuscript that addresses the points raised during the review process.

Please submit your revised manuscript by Apr 12 2026 11:59PM. If you will need more time than this to complete your revisions, please reply to this message or contact the journal office at plosntds@plos.org. When you're ready to submit your revision, log on to https://www.editorialmanager.com/pntd/ and select the 'Submissions Needing Revision' folder to locate your manuscript file.

Please include the following items when submitting your revised manuscript:

* A letter that responds to each point raised by the editor and reviewer(s). You should upload this letter as a separate file labeled 'Response to Reviewers'. This file does not need to include responses to any formatting updates and technical items listed in the 'Journal Requirements' section below.

* A marked-up copy of your manuscript that highlights changes made to the original version. You should upload this as a separate file labeled 'Revised Manuscript with Track Changes'.

* An unmarked version of your revised paper without tracked changes. You should upload this as a separate file labeled 'Manuscript'.

If you would like to make changes to your financial disclosure, competing interests statement, or data availability statement, please make these updates within the submission form at the time of resubmission. Guidelines for resubmitting your figure files are available below the reviewer comments at the end of this letter.

We look forward to receiving your revised manuscript.

Kind regards,

Clarence Mang'era, PhD

Academic Editor

PLOS Neglected Tropical Diseases

Paul Mireji

Section Editor

PLOS Neglected Tropical Diseases

Shaden Kamhawi

co-Editor-in-Chief

PLOS Neglected Tropical Diseases

orcid.org/0000-0003-4304-636XX

Paul Brindley

co-Editor-in-Chief

PLOS Neglected Tropical Diseases

orcid.org/0000-0003-1765-0002

**Additional Editor Comments:**

Please strengthen the methodological clarity by specifying data sources, software workflows, and adding statistical significance tests for your key comparisons. Additionally, revise the manuscript to better contextualise the public health relevance of your findings and explicitly discuss the study's limitations.

**Journal Requirements:**

1) We ask that a manuscript source file is provided at Revision. Please upload your manuscript file as a .doc, .docx, .rtf or .tex. If you are providing a .tex file, please upload it under the item type u2018LaTeX Source Fileu2019 and leave your .pdf version as the item type u2018Manuscriptu2019.

2) Please upload all main figures as separate Figure files in .tif or .eps format. For more information about how to convert and format your figure files please see our guidelines:

https://journals.plos.org/plosntds/s/figures

**Reviewers' comments:**

Reviewer's Responses to Questions

**Key Review Criteria Required for Acceptance?**

As you describe the new analyses required for acceptance, please consider the following:

**Methods**

-Are the objectives of the study clearly articulated with a clear testable hypothesis stated?

-Is the study design appropriate to address the stated objectives?

-Is the population clearly described and appropriate for the hypothesis being tested?

-Is the sample size sufficient to ensure adequate power to address the hypothesis being tested?

-Were correct statistical analysis used to support conclusions?

-Are there concerns about ethical or regulatory requirements being met?

Reviewer #1: This manuscript presents an analysis of landmark-based geometric morphometrics and challenges the classical assumption that increasing the number of landmarks improves shape discrimination. The concept of “influential landmarks” is highly relevant to morphometric research, particularly in entomological taxonomy and vector surveillance. Although the manuscript offers a novel and valuable contribution that deserves publication, certain methodological details require further clarification and elaboration to strengthen the overall study.

Title

The current title, “Landmark-based geometric morphometrics: Influential landmarks,” effectively reflects the methodological focus of the study. However, could the authors consider revising or expanding the title to highlight its potential contribution to species discrimination in medically important or vector insects, to better align with the journal’s applied scope?

Abstract

The abstract effectively summarizes the overall scope of the study. Nevertheless, it could be strengthened by adding essential statistical results or numerical findings that support the main conclusions, ensuring the abstract fully reflects the study’s analytical depth.

Introduction

1. In lines 2–3, the introduction briefly mentions that species identification can be difficult for morphologically similar or cryptic taxa. However, it does not sufficiently address why successful discrimination of these species is biologically or epidemiologically important, particularly in the context of vector-borne disease transmission. To better align with the scope of PLOS Neglected Tropical Diseases, the authors should elaborate on the biological relevance and public health implications of accurately identifying cryptic or closely related vector species. For instance, how would improved morphological discrimination contribute to vector surveillance, disease risk mapping, or control strategies?

2. In lines 18–25, the authors attempt to describe the research gap related to the dimensionality of landmark-based analyses and refer to concepts such as first PCs, multidimensionality problems, and eigenvalues of the correlation matrix. However, these technical terms are introduced without sufficient explanation or context. Could the authors provide brief clarifications or background descriptions for these terms? For example, explain where the first principal components (PCs) come from, what the multidimensionality problem represents in geometric morphometrics, final shape variables, and how eigenvalues in the correlation matrix are used to assess redundancy among landmarks.

3. In the final sentence, lines 29–32, of the Introduction, the authors summarize their findings by stating that smaller subsets of carefully chosen landmarks can provide better taxonomic discrimination than full landmark sets. However, such a statement presents results or conclusions that belong more appropriately in the Results or Discussion section rather than in the Introduction. It would be more suitable here to focus on the potential benefits or significance of the study instead.

Materials and Methods

1. In lines 35–92, the manuscript does not clearly specify the sources of the morphometric data used in each analysis. It is unclear whether the landmark coordinates were newly digitized from specimens or re-used from previously published datasets or online repositories. However, i note that *Table 1 (“Materials. Genera and Species. , not mentioned; op, operator; F, females; M, males; peudow., pseudowillmori; sawad., sawadwongporni”) appears in the Results section. Given that the study analyzes images/data derived from previously published sources (i.e., secondary material), this table belongs more appropriately in the Materials and Methods section.

2. In lines 93–106, Several technical terms used in the Materials and Methods section, such as Procrustes superposition, resistant-fit (RF), allometric residue, random search method, and hierarchical method, are introduced without sufficient explanation or definition. For clarity and accessibility, it is recommended that the authors provide brief descriptions or conceptual explanations of these terms when they first appear. This will help readers—especially those less familiar with geometric morphometric techniques—understand the analytical framework and rationale behind each procedure more clearly.

3. The manuscript applies K-means clustering for group reclassification, rather than the more commonly used supervised methods such as Linear Discriminant Analysis (LDA) or Canonical Variate Analysis (CVA), which are standard in geometric morphometric studies. Could the authors clarify why an unsupervised classification approach (K-means) was chosen instead of a supervised one?

4. The manuscript indicates that all computations were performed using the XYOM software. However, it remains unclear whether XYOM can directly calculate residual allometry for subsequent correlation analyses, or if this step required additional processing using other software (e.g., R). Could the authors clarify whether XYOM itself was used to compute the allometric residuals, or if a separate platform or custom script was applied for this purpose? In addition, since the manuscript mentions that the resistant-fit alignment was performed using the R “opSup” function [21], please specify whether R was also used for other parts of the analysis (such as allometry correction or statistical correlation). Providing these details would enhance methodological transparency and reproducibility.

Reviewer #2: The objectives of the study are clearly articulated, the study design is appropriate, the population and sample size are suitable, and the statistical analyses are correctly applied to support the conclusions.

However, it would be helpful if the Materials and Methods section clarified the relationship between the “random search method” and the “hierarchical method”—specifically, whether it is necessary to perform one method before evaluating the other, or how exactly they are related in the workflow.

**********

**Results**

-Does the analysis presented match the analysis plan?

-Are the results clearly and completely presented?

-Are the figures (Tables, Images) of sufficient quality for clarity?

Reviewer #1: 1. The results section mainly presents percentages and correlation coefficients; however, there is no indication of statistical significance testing (e.g., p-values). Could the authors include appropriate significance tests to confirm whether the subsets of landmarks truly outperform the full landmark sets in a statistically significant way?

2. All tables could be improved for better readability, as they currently contain a large number of abbreviations that may make interpretation difficult. I recommend revising the tables—especially the column headers—by avoiding abbreviations and using full terms instead. This would make the presentation clearer and more reader-friendly across all tables.

Reviewer #2: The results are clearly and completely presented, and the figures, tables, and images are of sufficient quality and clarity. The analysis matches the stated analysis plan.

**********

**Conclusions**

-Are the conclusions supported by the data presented?

-Are the limitations of analysis clearly described?

-Do the authors discuss how these data can be helpful to advance our understanding of the topic under study?

-Is public health relevance addressed?

Reviewer #1: The Discussion and conclusion sections are excellently written — it explains the results clearly and effectively connects them to potential applications. However, I suggest adding a brief paragraph that highlights how these findings could be applied in vector surveillance and control programs, to better align the discussion with the journal’s scope and readership.

Reviewer #2: The conclusions of the study are well supported by the data presented in the manuscript. However, the limitations of the analysis are not clearly described in a dedicated section or paragraph. Including a concise summary of the main limitations would provide important context and enhance the transparency and interpretability of the study’s findings.

Regarding the broader impact, the manuscript discusses how these data contribute to advancing our understanding of the studied technique, particularly in identifying efficient subsets of landmarks for morphometric analysis. However, the public health relevance of this work is not clearly detailed. While the introduction briefly mentions potential relevance, no further discussion is provided on this aspect in the results or discussion sections.

**********

**Editorial and Data Presentation Modifications?**

Use this section for editorial suggestions as well as relatively minor modifications of existing data that would enhance clarity. If the only modifications needed are minor and/or editorial, you may wish to recommend “Minor Revision” or “Accept”.

Reviewer #1: -

Reviewer #2: (No Response)

**********

**Summary and General Comments**

Use this section to provide overall comments, discuss strengths/weaknesses of the study, novelty, significance, general execution and scholarship. You may also include additional comments for the author, including concerns about dual publication, research ethics, or publication ethics. If requesting major revision, please articulate the new experiments that are needed.

Reviewer #1: -

Reviewer #2: This manuscript presents a well-structured study on the efficiency of landmark subsets for morphometric discrimination among closely related insect taxa. The objectives are clearly stated, the study design and statistical procedures are well detailed, the sample sizes are sufficient, and the statistical analyses are correctly applied. The results are clearly presented, and the figures and tables are of good quality. The conclusions are well supported by the data.

Among the strengths of the study are the systematic comparison of the random search and hierarchical methods, and the demonstration that smaller, carefully selected landmark subsets can, in some cases, outperform the full set. However, the relationship between the two methods is not fully explained, and the limitations of the study are not explicitly presented. Additionally, while the technical utility of the findings is discussed, the public health relevance is only briefly mentioned and not explored in depth.

Overall, this is a novel and carefully executed study that advances our understanding of landmark selection in geometric morphometrics. Clarifying the relationship between the methods, explicitly stating the limitations, and contextualizing the relevance would further strengthen the manuscript.

**********

PLOS authors have the option to publish the peer review history of their article (what does this mean?). If published, this will include your full peer review and any attached files.

If you choose “no”, your identity will remain anonymous but your review may still be made public.

**Do you want your identity to be public for this peer review?** For information about this choice, including consent withdrawal, please see our Privacy Policy.

Reviewer #1: No

Reviewer #2: No

[NOTE: If reviewer comments were submitted as an attachment file, they will be attached to this email and accessible via the submission site. Please log into your account, locate the manuscript record, and check for the action link "View Attachments". If this link does not appear, there are no attachment files.]

**Figure resubmission:**

While revising your submission, we strongly recommend that you use PLOS’s NAAS tool (https://ngplosjournals.pagemajik.ai/artanalysis) to test your figure files. NAAS can convert your figure files to the TIFF file type and meet basic requirements (such as print size, resolution), or provide you with a report on issues that do not meet our requirements and that NAAS cannot fix.

After uploading your figures to PLOS’s NAAS tool - https://ngplosjournals.pagemajik.ai/artanalysis, NAAS will process the files provided and display the results in the "Uploaded Files" section of the page as the processing is complete. If the uploaded figures meet our requirements (or NAAS is able to fix the files to meet our requirements), the figure will be marked as "fixed" above. If NAAS is unable to fix the files, a red "failed" label will appear above. When NAAS has confirmed that the figure files meet our requirements, please download the file via the download option, and include these NAAS processed figure files when submitting your revised manuscript.

**Reproducibility:**

To enhance the reproducibility of your results, we recommend that authors of applicable studies deposit laboratory protocols in protocols.io, where a protocol can be assigned its own identifier (DOI) such that it can be cited independently in the future. Additionally, PLOS ONE offers an option to publish peer-reviewed clinical study protocols. Read more information on sharing protocols at https://plos.org/protocols?utm_medium=editorial-email&utm_source=authorletters&utm_campaign=protocols

Attachment Submitted filename: renamed_488df.docx

# Author comment: Author response to Decision Letter 1

**DOI:** 10.1371/journal.pntd.0014386.r002

16 Apr 2026

Attachment Submitted filename: Responses to reviewers.pdf

# Peer review: Decision Letter 1

**DOI:** 10.1371/journal.pntd.0014386.r003

**Authors:** Clarence Mang'era

15 May 2026

Dear Dr. dujardin,

We are pleased to inform you that your manuscript 'Landmark-based morphometrics with fewer landmarks: some examples for medical entomology.' has been provisionally accepted for publication in PLOS Neglected Tropical Diseases.

Before your manuscript can be formally accepted you will need to complete some formatting changes, which you will receive in a follow up email. A member of our team will be in touch with a set of requests.

Please note that your manuscript will not be scheduled for publication until you have made the required changes, so a swift response is appreciated.

IMPORTANT: The editorial review process is now complete. PLOS will only permit corrections to spelling, formatting or significant scientific errors from this point onwards. Requests for major changes, or any which affect the scientific understanding of your work, will cause delays to the publication date of your manuscript.

Should you, your institution's press office or the journal office choose to press release your paper, you will automatically be opted out of early publication. We ask that you notify us now if you or your institution is planning to press release the article. All press must be co-ordinated with PLOS.

Thank you again for supporting Open Access publishing; we are looking forward to publishing your work in PLOS Neglected Tropical Diseases.

Best regards,

Clarence Mang'era, PhD

Academic Editor

PLOS Neglected Tropical Diseases

Paul Mireji

Section Editor

PLOS Neglected Tropical Diseases

Shaden Kamhawi

co-Editor-in-Chief

PLOS Neglected Tropical Diseases

orcid.org/0000-0003-4304-636XX

Paul Brindley

co-Editor-in-Chief

PLOS Neglected Tropical Diseases

orcid.org/0000-0003-1765-0002

***********************************************************

p.p1 {margin: 0.0px 0.0px 0.0px 0.0px; line-height: 16.0px; font: 14.0px Arial; color: #323333; -webkit-text-stroke: #323333}span.s1 {font-kerning: none

Reviewer's Responses to Questions

**Key Review Criteria Required for Acceptance?**

As you describe the new analyses required for acceptance, please consider the following:

**Methods**

-Are the objectives of the study clearly articulated with a clear testable hypothesis stated?

-Is the study design appropriate to address the stated objectives?

-Is the population clearly described and appropriate for the hypothesis being tested?

-Is the sample size sufficient to ensure adequate power to address the hypothesis being tested?

-Were correct statistical analysis used to support conclusions?

-Are there concerns about ethical or regulatory requirements being met?

Reviewer #1: No further major methodological concerns remain. The authors have adequately explained the relevant methodological issues in the revised manuscript and have responded well to the recommendations provided. The objectives, data sources, analytical approach, statistical rationale, and software workflow are now sufficiently described. The revisions also improve the clarity and reproducibility of the study, particularly regarding the use of reduced landmark subsets, adjusted reclassification scores, and the application of XYOM and R where appropriate.

Overall, the authors have addressed the methodological comments satisfactorily, and I have no additional concerns regarding the key review criteria required for acceptance.

Reviewer #2: The study presents clearly formulated research questions and objectives, accompanied by explicit and testable hypotheses. The experimental and analytical design is appropriate to address the stated objectives, allowing for an adequate evaluation of the relationships among the variables studied.

The samples used are clearly described, including selection criteria and relevant characteristics for the interpretation of the results. Furthermore, the sample size is adequate to support the analyses performed.

The study incorporates a detailed, robust, and novel statistical design, employing appropriate methodologies to support the conclusions obtained. The statistical analyses used are consistent with the proposed hypotheses and strengthen the validity of the presented results.

**********

**Results**

-Does the analysis presented match the analysis plan?

-Are the results clearly and completely presented?

-Are the figures (Tables, Images) of sufficient quality for clarity?

Reviewer #1: No further major methodological concerns remain. The authors have adequately explained the relevant methodological issues in the revised manuscript and have responded well to the recommendations provided. The objectives, data sources, analytical approach, statistical rationale, and software workflow are now sufficiently described. The revisions also improve the clarity and reproducibility of the study, particularly regarding the use of reduced landmark subsets, adjusted reclassification scores, and the application of XYOM and R where appropriate.

Overall, the authors have addressed the methodological comments satisfactorily, and I have no additional concerns regarding the key review criteria required for acceptance.

Reviewer #2: The analyses presented are consistent with the proposed analysis plan and appropriately address the objectives of the study. The results are clearly, comprehensively, and coherently presented, facilitating their interpretation. In addition, the figures, tables, and images are of sufficient quality and clarity, effectively supporting the presentation and understanding of the findings.

**********

**Conclusions**

-Are the conclusions supported by the data presented?

-Are the limitations of analysis clearly described?

-Do the authors discuss how these data can be helpful to advance our understanding of the topic under study?

-Is public health relevance addressed?

Reviewer #1: No further major methodological concerns remain. The authors have adequately explained the relevant methodological issues in the revised manuscript and have responded well to the recommendations provided. The objectives, data sources, analytical approach, statistical rationale, and software workflow are now sufficiently described. The revisions also improve the clarity and reproducibility of the study, particularly regarding the use of reduced landmark subsets, adjusted reclassification scores, and the application of XYOM and R where appropriate.

Overall, the authors have addressed the methodological comments satisfactorily, and I have no additional concerns regarding the key review criteria required for acceptance.

Reviewer #2: The conclusions are well supported by the data presented and are consistent with the results obtained in the study. The limitations of the analyses are clearly described and appropriately discussed by the authors. In addition, the manuscript includes a specific section dedicated to the epidemiological importance of the study

**********

PLOS authors have the option to publish the peer review history of their article (what does this mean?). If published, this will include your full peer review and any attached files.

If you choose “no”, your identity will remain anonymous but your review may still be made public.

**Do you want your identity to be public for this peer review?** For information about this choice, including consent withdrawal, please see our Privacy Policy.

Reviewer #1: No

Reviewer #2: No

# Editor report: Acceptance letter

**DOI:** 10.1371/journal.pntd.0014386.r004

**Authors:** Clarence Mang'era

Dear Dr. dujardin,

We are delighted to inform you that your manuscript, "Landmark-based morphometrics with fewer landmarks: some examples for medical entomology.," has been formally accepted for publication in PLOS Neglected Tropical Diseases.

We have now passed your article onto the PLOS Production Department who will complete the rest of the publication process. All authors will receive a confirmation email upon publication.

The corresponding author will soon be receiving a typeset proof for review, to ensure errors have not been introduced during production. Please review the PDF proof of your manuscript carefully, as this is the last chance to correct any scientific or type-setting errors. Please note that major changes, or those which affect the scientific understanding of the work, will likely cause delays to the publication date of your manuscript. Note: Proofs for Front Matter articles (Editorial, Viewpoint, Symposium, Review, etc...) are generated on a different schedule and may not be made available as quickly.

Soon after your final files are uploaded, the early version of your manuscript will be published online unless you opted out of this process. The date of the early version will be your article's publication date. The final article will be published to the same URL, and all versions of the paper will be accessible to readers.

For Research Articles, you will receive an invoice from PLOS for your publication fee after your manuscript has reached the completed accept phase. If you receive an email requesting payment before acceptance or for any other service, this may be a phishing scheme. Learn how to identify phishing emails and protect your accounts at https://explore.plos.org/phishing.

Thank you again for supporting open-access publishing; we are looking forward to publishing your work in PLOS Neglected Tropical Diseases.

Best regards,

Shaden Kamhawi

co-Editor-in-Chief

PLOS Neglected Tropical Diseases

Paul Brindley

co-Editor-in-Chief

PLOS Neglected Tropical Diseases
